# Concurrent Validity of Motion Parameters Measured With an RGB-D Camera-Based Markerless 3D Motion Tracking Method in Children and Young Adults

**DOI:** 10.1109/JTEHM.2024.3435334

**Published:** 2024-07-29

**Authors:** Nikolas Hesse, Sandra Baumgartner, Anja Gut, Hubertus J. A. Van Hedel

**Affiliations:** Swiss Children’s RehabUniversity Children’s Hospital Zurich 8910 Affoltern am Albis Switzerland; Children’s Research CenterUniversity Children’s Hospital Zurich, University of Zurich 8032 Zürich Switzerland

**Keywords:** Children, motion analysis, motion tracking, Kinect, RGB-D

## Abstract

Objective: Low-cost, portable RGB-D cameras with integrated motion tracking functionality enable easy-to-use 3D motion analysis without requiring expensive facilities and specialized personnel. However, the accuracy of existing systems is insufficient for most clinical applications, particularly when applied to children. In previous work, we developed an RGB-D camera-based motion tracking method and showed that it accurately captures body joint positions of children and young adults in 3D. In this study, the validity and accuracy of clinically relevant motion parameters that were computed from kinematics of our motion tracking method are evaluated in children and young adults. Methods: Twenty-three typically developing children and healthy young adults (5-29 years, 110–189 cm) performed five movement tasks while being recorded simultaneously with a marker-based Vicon system and an Azure Kinect RGB-D camera. Motion parameters were computed from the extracted kinematics of both methods: time series measurements, i.e., measurements over time, peak measurements, i.e., measurements at a single time instant, and movement smoothness. The agreement of these parameter values was evaluated using Pearson’s correlation coefficients r for time series data, and mean absolute error (MAE) and Bland-Altman plots with limits of agreement for peak measurements and smoothness. Results: Time series measurements showed strong to excellent correlations (r-values between 0.8 and 1.0), MAE for angles ranged from 1.5 to 5 degrees and for smoothness parameters (SPARC) from 0.02-0.09, while MAE for distance-related parameters ranged from 9 to 15 mm. Conclusion: Extracted motion parameters are valid and accurate for various movement tasks in children and young adults, demonstrating the suitability of our tracking method for clinical motion analysis. Clinical Impact: The low-cost portable hardware in combination with our tracking method enables motion analysis outside of specialized facilities while providing measurements that are close to those of the clinical gold-standard.

## I. Introduction

Clinical motion analysis plays an important role in the evaluation of patients’ motor functions and activities, in therapy planning and adaptation, and in monitoring progress over time [1]. In clinical practice, such analysis is often carried out in the form of observer-based assessments, which generally consist of a list of movement tasks and associated scoring criteria, based on which a therapist rates the patient’s function, capacity, or performance. As the scoring is based on the therapist’s subjective perception, it is subject to intra- and inter-rater variability. Furthermore, the therapists require training and sometimes certification, and the assessments rely on an ordinal scale, meaning that patients have to make substantial progress to improve their score [2]. To overcome these drawbacks, clinical assessments have been supplemented with technology, e.g., marker-based 3D motion analysis (3DMA), which is considered the gold standard for clinical gait analysis as it provides excellent accuracy in motion tracking [3]. Such quantitative measurements facilitate clinical decision-making, e.g., the need for neuro-orthopedic surgery, and the evaluation of the effectiveness of interventions [4]. While 3DMA is routinely applied to analyze gait, several other applications have been explored [5], e.g., assessing upper limb function [6], [7] or trunk control [8]. Nevertheless, various factors hinder the routine implementation of 3DMA for objective, fine-grained, interval-scaled therapeutic assessments. The accurate placement of markers on a patient’s body is time-consuming and requires the patients to cooperate, which may be especially difficult in the case of children with neurological impairments [9]. In addition, the high cost and spatial requirements restrict the use of these systems to specialized facilities, and the evaluation and interpretation of the findings require trained, experienced personnel [10].

These limitations could be overcome by using RGB-Depth (RGB-D) cameras with integrated 3D body tracking functionality. These cameras are portable, affordable, and do not require additional expertise or effort, like correct marker placement. Consequently, several studies have investigated the validity of different versions of the Microsoft Kinect camera [11], e.g., for postural control [12], walking, standing, and sitting [13], [14], upper limb function [15], and other tasks [16], [17], [18]. However, the validity of the captured kinematics appears to be limited [11], [13], [16], [17], task-dependent, [14], [15], and too low for clinical applications [17], [18]. To exploit the potential of RGB-D cameras to assess motor functions, we developed a custom method for markerless full-body motion tracking of children and adults from RGB-D sequences [19]. Code is available at https://github.com/nh236/smplify-kids. This method is based on previous work on infant motion tracking [20] and a method for estimating body shape and pose from images [21]. In an earlier study, we found the estimated *3D body joint positions* determined with our method were highly accurate and strongly agreed with a reference 3DMA system in 5 to 29-year-old healthy participants [19]. However, for clinical applications, kinematic parameters such as maximum joint angles or qualitative measures like movement smoothness, are more relevant than joint positions, and small errors in joint positions can still cause significant errors in other parameters. Therefore, the main objective of this study was to evaluate the concurrent validity of clinically relevant *motion parameters* extracted from the previously collected data set [19]. We analyzed the agreement between parameters computed from kinematics captured with our method and the Vicon system. Additionally, we evaluated the validity of parameters computed from the publicly available Azure Kinect Body Tracking (K4ABT) [22].

## II. Methods

### A. Participants

Twenty-three typically developing children and young adults (14 females, 9 males) were recruited through purposive sampling to include a variety of ages (5 – 29 years, mean 13.2 years) and body sizes (110 – 189 cm). Exclusion criteria were neurological, musculoskeletal, or cardiovascular diagnoses. Parents and participants were informed verbally and in writing and had to agree verbally before participating. Parents and participants aged 14 years and older additionally provided written informed consent. Participants could withdraw from the study at any time. The Ethical Committee of the Canton of Zurich approved this study (BASEC-Nr. PB_2016-01843).

### B. Movement Tasks

We selected five tasks involving different body parts that we adopted from clinical assessments of motor performance or that are challenging to track, e.g., due to self-occlusions and fast movements:

• Reach to the other side: The participant reaches across body midline to roughly shoulder height, returns to starting position, and repeats task with the other arm.
• Trunk bending: The participant bends the trunk to the left, right, forward, and backward.
• Standing straight leg raise (SSLR): Participant raises one straight leg and holds for three seconds, then repeats with the other leg.
• Squats: The participant performs three squats with arms stretched out to the front.
• Jumping jacks: The participant performs three jumping jacks.

The tasks were explained to the participants and each task was demonstrated once. The starting position was upright standing, with arms hanging down.

### C. Data Collection

The participants were recorded using a Vicon system with 12 Vero V2.2 cameras at 120 Hz, except for two participants who were recorded at 90 Hz. Fifty-one markers (16 mm diameter) were placed according to the Conventional Gait Model 2.5 [23], covering the whole body, including the arms and head. Marker placement was carried out jointly by two experienced members of our gait lab, who routinely conduct clinical gait analyses. In addition, RGB-D data were simultaneously recorded at 30 Hz with the *Azure Kinect Developer Kit* (AKDK) in “NFOV unbinned” mode at a depth resolution of $640\times 576$ and colour image resolution of $1920\times 1080$. The AKDK was mounted on a tripod facing the participant frontally at a distance at which the entire body was visible (1.5 - 2.9 meters). The participants performed the tasks once with and a second time without markers (and the Vicon system). Both camera systems were temporally synchronized to avoid interference by the active illumination of the Vicon with the AKDK depth measurements. At the time of writing, Microsoft has discontinued AKDK, but the same technology is available from another vendor [24], including the body tracking component [25].

### D. Data Processing

#### 1) Vicon

Marker trajectories were post-processed with Vicon Nexus 2.10. The joint centers for the head, shoulders, elbows, wrists, hips, knees, ankles, and feet, as well as the center of mass were extracted.

#### 2) Our Method [19]

3D point clouds were computed from AKDK depth images and the camera calibration, using the Azure Kinect SDK [26]. These point clouds served as input to our method, which estimated pose and shape parameters of a parametric model of the body surface, termed *SMPL-H* [27], so that the virtual body matched the person in the input data [19]. The SMPL-H body model has an underlying skeleton, from which we extracted joint positions corresponding to those of the Vicon system. In a previous study, we evaluated the validity of our method with respect to Vicon on the same data set at the level of joint positions. We found that the mean per joint position error over all joints was 11.7 mm [19]. Pearson correlation coefficients were >0.95 for non-stationary joints (as opposed to stationary ones, such as ankle joints during reaching). More than 98% of the joints had an error below 5 cm, and tracking failures only occurred when the body occluded entire limbs. For more details, we refer the reader to [19].

#### 3) K4ABT

Joint positions were extracted from the recorded RGB-D sequences using the official Azure Kinect Sensor SDK [26] and K4ABT (version 1.1.2) [22].

Joint positions of our method and K4ABT were transformed to the Vicon coordinate frame as in [19]. To avoid introducing errors from different definitions of angle computations, angles were calculated as scalar values from joint positions for all systems similarly, e.g., the elbow angle was calculated from the shoulder, elbow, and wrist joint positions. We did not apply smoothing or filtering to the kinematics for any method.

### E. Motion Parameters

Evaluation criteria in observer-based assessments often do not provide clearly defined quantitative values, but rather rough, verbal descriptions. For example, for the “reach across midline” task in the Trunk Control Measurement Scale [28], the options are “child reaches target without difficulties”, “child reaches target, but has difficulties in performance”, or “child falls/cannot reach target”. Experienced physiotherapists formulated quantitative motion parameters based on such verbal descriptions, e.g., trunk rotation, elbow angle, maximum reach distance, and movement smoothness. We then computed these parameters from the captured kinematics. To provide a comprehensive evaluation, we include time series and peak measurements, because if one parameter type shows good results, it does not necessarily mean the other will, too.

*Time series measurements*

• *Trunk rotation* (during *reach* task): angle between line connecting hip joints and line connecting shoulder joints, projected to the horizontal plane.
• *Angle* parameters (*reach* and *squat*): angle between three joints, measured in each frame, e.g., shoulder, elbow and wrist joints for elbow angle.
• *Weight shift* (*SSLR*): 3D displacement of the center of mass (CoM).
• *Upper trunk displacement* (*squat*): 3D displacement of the midpoint between the two shoulder joints.
• *Speed*(*jumping jacks*): derivative of 3D joint positions (wrists and ankles).

*Peak measurements and smoothness of the movement*

• *Maximum reach distance* (*reach*): maximum wrist displacement with respect to the sagittal plane, which was calculated from the shoulder joints in the starting position.
• *Maximum trunk angles*(*trunk bending*) were computed with respect to the frontal and sagittal plane, which we defined based on shoulder joints and the “gravity” vector (from estimated ground plane).
• *Jump height* (*jumping jacks*): overall maximum distance between ankle position and the estimated ground plane.
• *Smoothness*(*all tasks except squat*) is related to the continuity and non-jerkiness of movements [29], e.g., the absence of abrupt changes. We quantified smoothness with the modified Spectral Arc length (SPARC), which decomposes a movement speed profile into higher- and lower-frequency components using the Fourier transform. It then computes a smoothness value using the assumption that smooth movements mostly contain low-frequency components, i.e., slow movements, and less smooth movements contain more high-frequency components, i.e., fast movements [27]. We computed SPARC values from the start to the end of specific movements, which we manually annotated, e.g., from the onset of arm motion to the maximum reach position. For rhythmic movements (*jumping jacks*), we split the sequence, calculated SPARC values per repetition, and averaged these, as recommended in [30].

### F. Statistics

**FIGURE 1.**
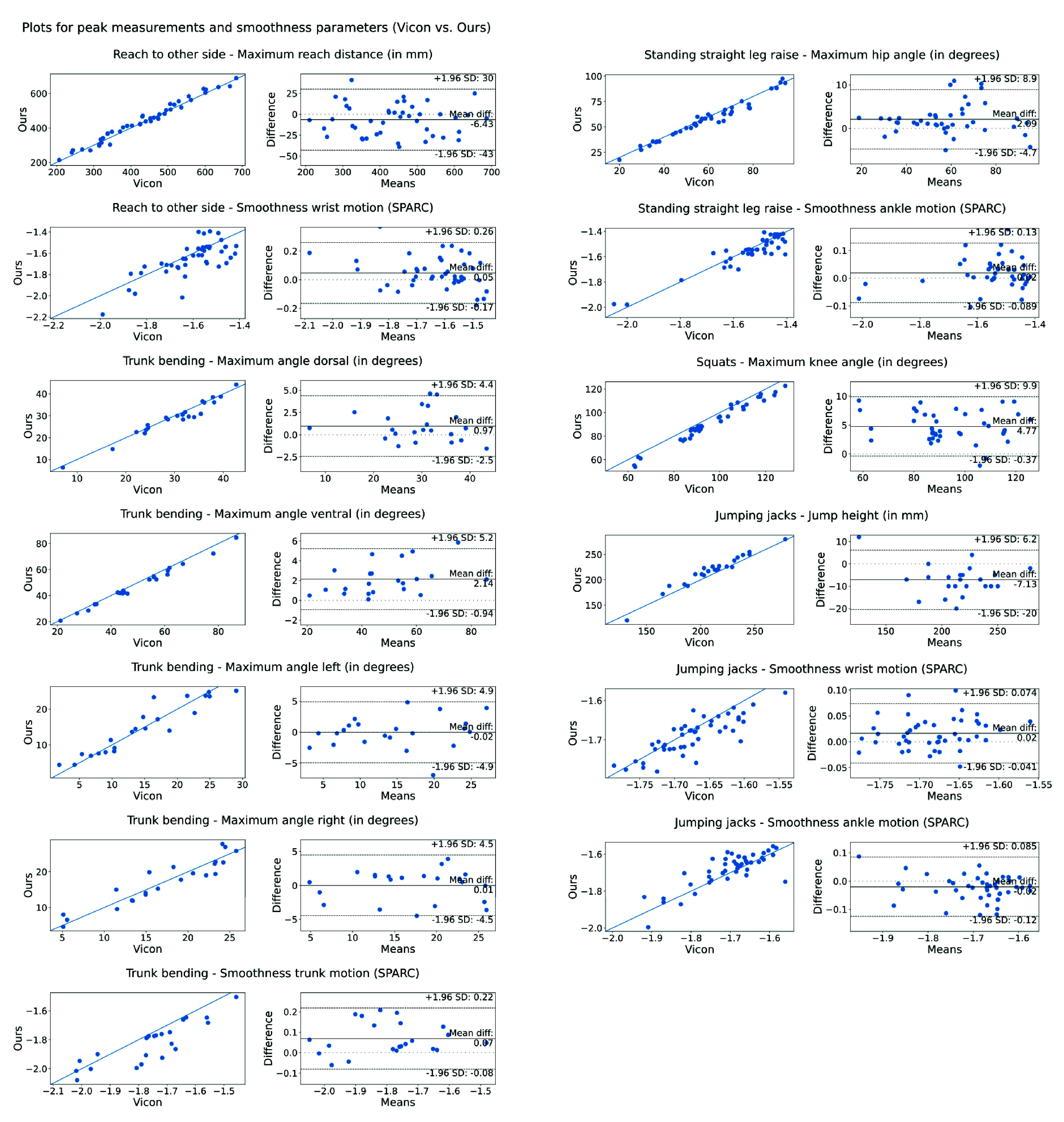
Scatter plots with line of equality (left sides) and Bland-Altman plots (right sides) for Ours vs. Vicon for peak measurements and smoothness parameters (corresponding to Table 2). Note that for improved visibility, the aspect ratio of the scatter plots (left sides) is not 1:1.

To evaluate the agreement between the parameters measured with two systems, we calculated Pearson’s correlation coefficient *r* for time series measurements. For peak measurements and smoothness values, we calculated the *mean absolute error* (MAE), i.e., the difference between the methods, and the *mean* of the measured values for each method. Additionally, we created scatterplots including the line of equality (*x = y*) and Bland-Altman plots (Fig. 1). Bland-Altman plots display the mean difference between the methods (y-axis) against their mean (x-axis). The average difference, i.e., the *bias*, indicates the systematic difference between the methods, while the bias $\pm 1.96\cdot $SD should include 95% of the differences in case the distribution is normal. The 95% confidence interval is also referred to as the limits of agreement (LoA), where bias $+ 1.96\cdot $SD is the *upper LoA* and bias - $1.96\cdot $SD is the *lower LoA*. Motion parameter calculation and statistical analysis were implemented in Python using the numpy package.

## III. Results

Twenty-three participants performed the five movement tasks. Due to technical problems, we had to exclude 8 recordings of three participants *with* markers, resulting in 107 recordings. Two participants were only recorded *with* markers, resulting in 105 recordings *without* markers. We excluded frames for which no body was detected by K4ABT from the evaluation (K4ABT vs. Ours). *Time series measurements* are displayed in Table 1, and *peak* and *smoothness parameters* in Table 2. In the following, we will refer to parameters computed from kinematics that were captured with our method as *Ours*, with the Vicon system as *Vicon*, and with K4ABT as *K4ABT*.

**TABLE 1:** Pearson Correlation Coefficients for Different Time Series Measurements

Task	Parameter	Ours vs. Vicon Pearson’s r	Ours vs. Vicon MAE (SD)	K4ABT vs. Ours Pearson’s r	K4ABT vs. Ours MAE (SD)
Reach	Trunk rotation	0.98 (0.05)	5.17° (2.07°)	0.82 (0.17)	6.75° (3.27°)
	Elbow angle	0.95 (0.08)	4.93° (1.75°)	0.83 (0.18)	8.69° (5.77°)
Standing straight leg raise	Weight shift CoM (medio-lateral)	1.00 (0.00)	2.65 mm (1.39 mm)	N/A	N/A
	Weight shift CoM (vertical)	0.96 (0.03)	2.5 mm (0.81 mm)	N/A	N/A
	Weight shift CoM (anterio-posterior)	0.99 (0.01)	2.0 mm (1.1 mm)	N/A	N/A
Squat	Ankle angle	0.80 (0.25)	6.24° (3.05°)	0.82 (0.15)	6.2° (3.08°)
	Knee angle	1.00 (0.00)	3.49° (1.26°)	0.99 (0.01)	3.86° (1.99°)
	Hip angle	0.99 (0.02)	2.08° (0.71°)	0.98 (0.03)	3.66° (2.27°)
	Upper trunk displacement (medio-lateral)	0.96 (0.04)	2.35 mm (1.01 mm)	0.82 (0.25)	7.1 mm (8.18 mm)
	Upper trunk displacement (vertical)	1.00 (0.01)	6.8 mm (3.11 mm)	0.99 (0.01)	11.19 mm (6.74 mm)
	Upper trunk displacement (anterio-posterior)	0.96 (0.04)	6.1 mm (3.1 mm)	0.88 (0.16)	17.1 mm (19.63 mm)
Jumping jacks	Wrist speed	0.99 (0.01)	1.16 m/s (0.35 m/s)	0.92 (0.10)	2.47 m/s (1.21 m/s)
	Ankle speed	0.98 (0.02)	0.64 m/s (0.1 m/s)	0.73 (0.22)	1.99 m/s (1.34 m/s)

Average Pearson’s correlation coefficient and mean absolute error (MAE) calculated over all participants, standard deviation in brackets. For angles one-dimensional, for 3D displacement in medio-lateral, vertical, and anterio-posterior directions. p-values were <0.001 except for Ankle angles over time (Ours vs. Vicon, 0.0016) and Upper trunk displacement over time medio-lateral (Ours vs. K4ABT, 0.028). CoM: Center of Mass. N/A: K4ABT does not provide center of mass.

**TABLE 2:** Mean Absolute Differences (Mean Absolute Error) in Measured Values for Ours vs. Vicon and K4ABT vs. Ours

Task	Parameter	Ours vs. Vicon					K4ABT vs. Ours				
		Mean Ours	Mean Vicon	Bias (p-value)	$1.96~\cdot $ SD	MAE Ours vs. Vicon	Mean K4ABT	Mean Ours	Bias (p-value)	$1.96~\cdot $ SD	MAE K4ABT vs. Ours
Reach	Max. reach distance (mm)	442.55 (117.98)	436.11 (117.18)	6.432 (0.015)	36.454	15.32 (10.87)	416.67 (113.64)	441.71 (129.20)	−25.88 (<0.001)	74.594	39.57 (24.51)
	Smoothness wrist motion	−1.65 (0.16)	−1.61 (0.13)	−0.047 (0.007)	0.212	0.09 (0.08)	−1.99 (0.30)	−1.65 (0.16)	−0.335 (<0.001)	0.506	0.34 (0.26)
Trunk	Max. angle dorsal (degrees)	29.00 (8.13)	29.96 (7.96)	−0.966 (0.017)	3.420	1.48 (1.34)	25.48 (7.14)	26.73 (6.14)	−1.243 (0.47)	13.348	3.90 (5.72)
	Max. angle ventral (degrees)	47.67 (14.99)	49.82 (15.63)	−2.142 (<0.001)	3.079	2.14 (1.57)	48.28 (16.10)	46.55 (12.32)	1.733 (0.24)	12.330	3.11 (5.74)
	Max. angle left (degrees)	14.65 (6.97)	14.63 (7.41)	0.023 (0.943)	4.915	1.82 (1.73)	13.24 (5.32)	15.64 (5.87)	−2.4 (<0.001)	5.768	3.15 (2.12)
	Max. angle right (degrees)	17.08 (6.41)	17.08 (6.43)	−0.005 (0.973)	4.478	1.93 (1.23)	15.94 (5.73)	16.97 (4.62)	−1.03 (0.04)	6.963	3.00 (2.16)
	Smoothness trunk motion	−1.83 (0.14)	−1.76 (0.15)	−0.069 (<0.001)	0.149	0.08 (0.07)	−1.93 (0.14)	−1.80 (0.14)	−0.129 (<0.001)	0.172	0.14 (0.08)
SSLR	Max. hip angle (degrees)	57.32 (18.23)	59.35 (18.39)	−2.093 (<0.001)	6.835	2.86 (2.83)	61.09 (17.14)	62.18 (16.30)	−1.095 (0.36)	19.651	5.12 (8.69)
	Smoothness ankle motion	−1.56 (0.13)	−1.54 (0.15)	-0.019 (0.035)	0.107	0.04 (0.04)	−1.86 (0.35)	−1.56 (0.21)	−0.292 (<0.001)	0.515	0.29 (0.26)
Squat	Max. knee angles (degrees)	92.10 (17.10)	96.78 (16.79)	−4.768 (<0.001)	5.135	5.04 (2.49)	96.70 (14.70)	99.14 (16.74)	−2.44 (0.04)	14.858	6.23 (5.30)
Jumping Jacks	Jump height (mm)	217.65 (31.88)	210.70 (30.03)	7.130 (<0.001)	13.322	8.83 (4.46)	220.05 (69.73)	204.19 (23.98)	15.857 (0.24)	119.109	30.19 (55.14)
	Smoothness wrist motion	−1.69 (0.05)	−1.67 (0.05)	−0.016 (<0.001)	0.057	0.02 (0.02)	−1.72 (0.08)	−1.69 (0.05)	−0.034 (<0.001)	0.184	0.07 (0.07)
	Smoothness ankle motion	−1.68 (0.09)	−1.70 (0.08)	0.020 (0.16)	0.105	0.04 (0.04)	−1.81 (0.13)	−1.68 (0.08)	−0.132 (<0.001)	0.263	0.15 (0.11)

Mean over measured values. Average over all participants, standard deviation in brackets. Note that “Ours vs. Vicon” and “K4ABT vs. Ours” were evaluated on different sequences of the same participants (with and without markers). Due to the large variability in task performance, especially for younger children, mean values between the two evaluations (“Ours vs. Vicon” and “K4ABT vs. Ours”) should not be compared directly. Note that MAE is the meanabsolute difference, and therefore not the same as the difference between the mean values. P-values for bias computed with the paired t-test.

### A. Ours vs. Vicon

**FIGURE 2.**
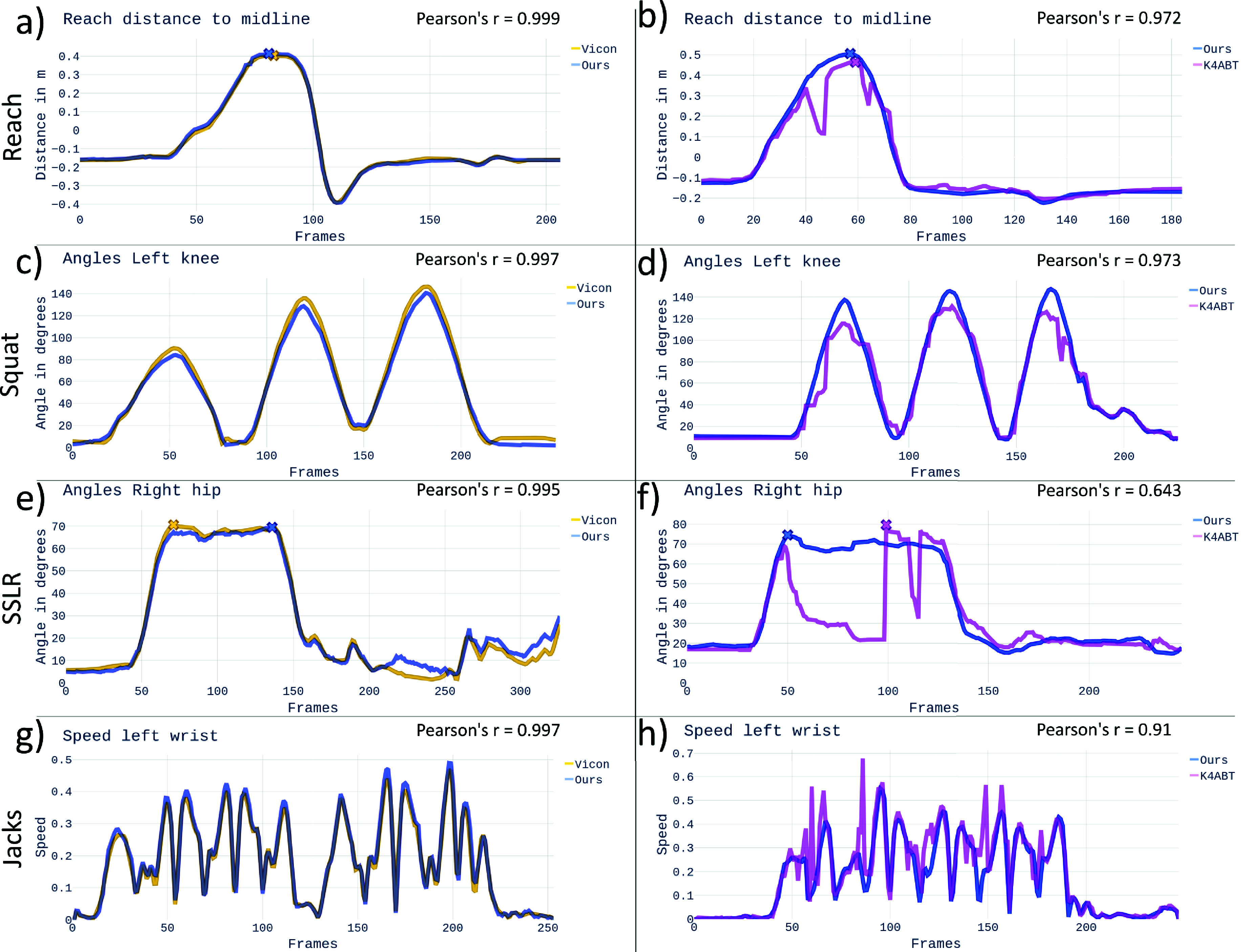
Sample plots of time series measurements. Each row represents measurements of the same participant. Left: Ours (blue) vs. Vicon (yellow). Right: K4ABT (pink) vs. Ours (blue). a) and b): reach to the other side: reach distance with respect to body midline, x marking maximum reach distance. c) and d): squat: knee angles. e) and f): SSLR: hip angles, x denotes maximum angle. g) and h): jumping jacks: wrist speed. While parameters measured with our method highly agree with Vicon results (left), we observed repeated tracking failures for K4ABT (right), e.g., tracking of the arm lost during reaching (b), inaccurate knee angles during squat (d), tracking of the leg lost during leg raise (f) and hand tracking failures during jumping jacks (h). Note that the results on the left and the right sides are from different recordings of the same participant, once with markers (left) and once without markers (right). Best viewed in colour. Corresponding motion sequences are shown in the supplementary video.

The *time series measurements* correlated highly between Ours and Vicon (Table 1), with $r \ge 0.95$ for all angles, except for ankle angles during the squat ( $r =0.80$). Correlations of displacements were very high ( $r > 0.99$) for directions with the highest amount of motion, i.e., medio-lateral for the center of mass during SSLR and vertical trunk displacement for the squat, and slightly lower for other directions ( $r > 0.95$). Speed parameters had *r*-values $\ge 0.98$. As displayed in Table 2 and Fig. 1 *peak measurements* of positional data obtained an average MAE between 8.8 and 15.3 mm, and *1.96* $\cdot $*SD* between 13.3 and 36.5 mm. Regarding angles, the average MAE ranged from 1.5 to 5 degrees, and *1.96* $\cdot $*SD* ranged from 3 to 6.8 degrees. MAE and *1.96* $\cdot $*SD* of SPARC smoothness values ranged from 0.02 to 0.09 and 0.057 to 0.212, respectively. Most parameters showed a small, yet statistically significant, bias, and some maximum angles were slightly underestimated in Ours compared to Vicon (dorsal and ventral trunk angles, knee angles during squat). Jump height was slightly overestimated and SPARC values were consistently marginally lower in Ours, i.e., less smooth. Examples for the agreement between Ours and Vicon for single participants are visualized in Fig. 2, left side.

### B. K4ABT vs. Ours

Due to marker interference, K4ABT results should not be compared to the Vicon system directly [19]. To give an impression of K4ABT capabilities, we compared K4ABT to Ours, but, since our method cannot be considered a gold standard method, we only evaluated results on a coarse level. Correlations were nearly perfect for the knee and hip angles and the vertical trunk displacement over time during the squat (Table 1, right). Other joint angles and displacement time series measurements showed smaller *r*-values (0.82 – 0.88), while speed had higher correlations for wrists ( $r =0.92$) than for ankles ( $r =0.73$).

Peak measurements for K4ABT vs. Ours (Table 2, right side) showed consistently higher error levels than for Ours vs. Vicon, but within acceptable ranges, and less smooth signals, i.e., lower SPARC values. These findings are reflected in visualizations of parameters for single participants in Fig. 2, where K4ABT measurements (Fig. 2, right side, pink) show relatively large jumps in some parts of the sequence, while the values agree very well for other parts.

## IV. Discussion

We found excellent agreement between Ours and Vicon, with most *r*-values >0.95. Positional and angle errors, as well as differences in smoothness values were small (MAE <16 mm and $\le 5$ degrees, SPARC MAE $\le 0.1$, respectively). Most parameters only showed a small (yet statistically significant) bias, and the *1.96* $\cdot $*SD* ranges were narrow. This indicates that our method is valid for extracting motion parameters for quantitative motion analysis.

Ankle angles during the squat showed the lowest correlations of all parameters ( $r =0.8$). A visual examination of tracking results revealed that in few sequences, marker distortions caused deformations of the lower leg in the point cloud, which led to less accurate fitting of the body model and resulted in less accurate ankle angles for Ours (see supplementary video). We did not observe this issue in sequences without markers.

A recent study evaluated a commercial markerless multi-camera system, “The Captury” [31], with respect to a Vicon system for fourteen children (3-6 years) performing squats and standing broad jumps [32]. Results from [32] for parameters most similar to those evaluated in the present study were: Maximum knee flexion during squat with a bias of -11.7 degrees and *1.96* $\cdot $*SD* of 11.9 degrees (Ours: bias of 4.8 degrees, *1.96* $\cdot $*SD* of 5.1 degrees). Jump height during broad jump had a bias of -8 mm and *1.96* $\cdot $*SD* of 30 mm (for maximum jump height during jumping jacks, our method had a bias of 7.1 mm and *1.96* $\cdot $*SD* of 13.3 mm). While these values are not directly comparable to our results due to differences in age groups and tasks, we consider our results to lie in similar (or even lower) error ranges as the commercial multi-camera system.

The degree of accuracy considered “good enough” depends on the targeted application. For comprehensive gait analysis, on which surgical decisions are based, error levels of less than two degrees are considered acceptable, and those below five degrees are reasonable [33]. Physiotherapists, on the other hand, can only accurately detect angle changes during slow, single-plane movements that are above 12 degrees [34]. Our method does not aim to replace marker-based systems in applications requiring the highest accuracy, but rather enhance observer-based assessments that involve visual estimates of joint angles or other qualitative aspects of movement, such as smoothness. Our method is best applicable in assessment-like, controlled settings, with patients being relatively stationary, i.e., standing or sitting, while facing the camera, and following standardized protocols. It is less feasible to assess patients who move freely in unrestricted environments. The low cost and portability of the hardware enable motion measurements outside the lab or in situations where marker-based methods are not applicable, e.g., robot-assisted gait therapy [35].

### A. Comparison K4ABT vs. Ours

The evaluation of K4ABT with our method as a reference showed that some parameters highly agree between both systems, while others show less agreement. SPARC values were consistently lower, i.e., less smooth, in K4ABT compared to Ours. This is caused by the overall lower accuracy of K4ABT but also by tracking failures that led to jumps in measurements (see Fig. 2).

One finding from our previous validation study is not clearly reflected in the evaluation of motion parameters: Nearly two-thirds of sequences contained short periods of tracking errors for K4ABT [19]. In the present study, we mainly observed good correlations and minor differences between K4ABT and Ours. Despite losing track of limbs during movement phases, K4ABT often recovered in the peak position, leading to relatively correct values for these parameters (see Fig. 2). However, it cannot be guaranteed that the tracking result is valid at the time of measurement. This is in line with previous studies, which concluded that the overall agreement of K4ABT is acceptable, but its accuracy is limited, and tracking errors occur repeatedly [12], [16].

### B. Limitations

As in our previous validation study [19], markers of the reference system directly influence our method’s results and, therefore, the calculated motion parameters. In some situations, this becomes evident, e.g., regarding ankle angles during the squat for some participants, while the effect for most other parameters is small. In the targeted application, participants will be recorded without markers, which is why we consider the presented error values to be an upper limit for the expected error.

We included only healthy participants in this study because all participants should be able to execute all five tasks. To show that our method generalizes across ages and sizes, we evaluated all participants together. In the results, we did not observe differences between children and adults. Further studies are needed to validate the method in patients.

We selected movement tasks that covered all body parts and posed challenges for motion tracking methods. Some did not have clearly defined quantitative evaluation criteria associated with them, but rather verbal/descriptive scoring criteria, which is why the computed quantitative motion parameters were chosen to reflect these descriptions. However, the results of this study and our previous validation study [19] suggest that the validity would stay the same for different parameter choices since our approach provides consistently accurate and valid measurements.

One limitation of our method is that the skeleton definition of the SMPL-H model [27] does not conform to the recommendations of the International Society of Biomechanics (ISB) on reporting human joint motion. The conversion of SMPL-H to follow the ISB definitions is not straightforward, and could introduce an additional source of errors that would make it harder to assess the capabilities of our method. For this reason, we evaluate “simple” parameters that might be insufficient for comprehensive gait analysis, but can support therapists during clinical assessments with objective, quantitative measurements. Recent work has added a biomechanical skeleton to an adult parametric body model [36] to resolve this limitation. To apply this model to children, we need to adapt the skeleton structure to the anthropometrics of smaller children.

## V. Conclusion

In this study, we evaluated the validity of our custom method for markerless full-body motion tracking for extracting clinically relevant motion parameters from RGB-D sequences of children and young adults for five different movement tasks. Parameters computed from our method were consistently close to those of the clinical reference standard system, confirming our previous study on validating our method at the level of joint positions [19]. We conclude that our method is valid for quantitative motion analysis in children and young adults.

This validation study is a step towards translating our method to clinical practice, where it can fill the gap between highly accurate 3DMA and routinely applied therapist-rated clinical assessments. By making the code of our tracking method publicly available, we want to motivate clinicians, engineers, and other researchers to use, validate, and extend the method to support the translation of easy-to-use, quantitative motion analysis into clinical routine. In ongoing studies, we are recording pediatric patients during clinical assessments of trunk control and upper limb function to demonstrate that the measurements taken with our method correspond to the observations of the therapists.

## Supplementary Materials

Supplementary materials
