# Supplementary material for: Concurrent Validity of Motion Parameters Measured With an RGB-D Camera-Based Markerless 3D Motion Tracking Method in Children and Young Adults
Source: IEEE J Transl Eng Health Med. 2024 Jul 29;12:580–8. doi: 10.1109/JTEHM.2024.3435334 (PMC11329219; doi:10.1109/JTEHM.2024.3435334)
Supplement: Supplementary materials [file supp2-3435334.docx]

Concurrent Validity of Motion Parameters Measured with an RGB-D Camera-based Markerless 3D Motion Tracking Method in Children and Young Adults

Nikolas Hesse, Sandra Baumgartner, Anja Gut, Hubertus JA van Hedel

Supplementary material


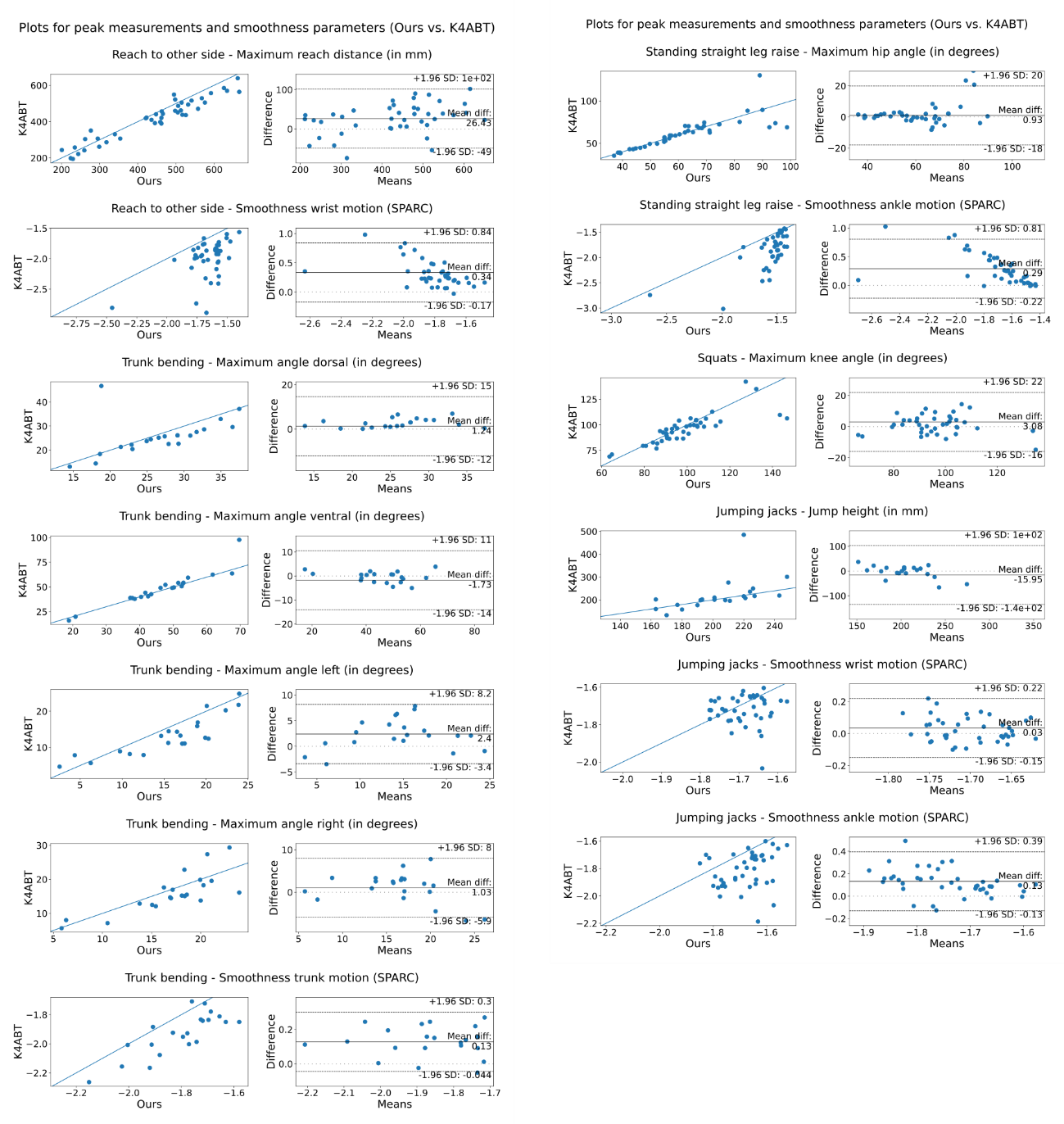


Figure 1: Scatter plots with line of equality (left sides) and Bland-Altman plots (right sides) for Ours vs. K4ABT for peak measurements and smoothness parameters (corresponding to Table 2 of main manuscript)

## SPARC definition [1]

SPARC builds on the assumption that a smooth movement is continuous and non-jerky, i.e., there are no abrupt movement changes. The goal is to divide the speed profile of the movement into slow and fast components, where the slow movements correspond to the targeted movement, and the fast movements correspond to jerkiness or tremor.

To compute a value that encapsulates this, the speed profile of a movement is decomposed into higher and lower frequency components using Fourier transform. The SPARC is then computed as the arc length of the Fourier magnitude spectrum.

The formula to compute the SPARC value is

$\text{SPARC =} -\int_{0}^{\omega_{c}} \left[ \left( \frac{1}{\omega_{c}} \right)^{2} + \left( \frac{d\hat{V}\left( \omega\right)}{d\omega} \right)^{2} \right]^{\frac{1}{2}}d\omega; \hat{V}\left( \omega\right)=\frac{V\left( \omega\right)}{V\left( 0 \right)}$,

where *V*(*ω*) is the Fourier magnitude spectrum *v*(*t*), $\hat{V}\left( \omega\right)$ is the normalized magnitude spectrum, normalized with respect to the DC magnitude *V*(0), and *ω_c_* is adaptively selected based on a given threshold $\overline{V}$ and is upper-bound by $\omega_{c}^{max}$:

$$\omega_{c} =\min\left\{ \omega_{c}^{max},\min\left\{ \omega,\hat{V}\left( r \right)<\overline{V} \forall r>\omega\right\} \right\}$$

## Center of Mass computation

We use the trimesh library [3] to compute the center of mass from a (watertight) SMPLH mesh. The trimesh implementation follows the algorithm specified in [4]

# References

| [1] | S. Balasubramanian, A. Melendez-Calderon, A. Roby-Brami and E. Burdet, "On the analysis of movement smoothness," *Journal of neuroengineering and rehabilitation,* vol. 12, p. 1–11, 2015. |
| --- | --- |
| [2] | S. Balasubramanian, A. Melendez-Calderon and E. Burdet, "A Robust and Sensitive Metric for Quantifying Movement Smoothness," *IEEE Transactions on Biomedical Engineering,* vol. 59, pp. 2126-2136, 2012. |
| [3] | Dawson-Haggerty et al., https:/trimesh.org, *trimesh,* 2019. |
| [4] | D. Eberly, *Polyhedral Mass Properties (Revisited), http://www.geometrictools.com/Documentation/PolyhedralMassProperties.pdf,* 2002. |
